# Landscape genomics analysis reveals the genetic basis underlying cashmere goats and dairy goats adaptation to frigid environments

**DOI:** 10.1007/s44154-025-00254-5

**Published:** 2025-09-09

**Authors:** Jianqing Zhao, Weiwei Yao, Qingqing Liu, Ping Gong, Yuanpan Mu, Wei Wang, Baolong Liu, Cong Li, Hengbo Shi, Jun Luo

**Affiliations:** 1 Shaanxi Key Laboratory of Molecular Biology for Agriculture, College of Animal Science and Technology, Northwest A&F University, Yangling, Shaanxi China; 2 Institute of Dairy Science, College of Animal Sciences, Zhejiang University, Hangzhou, China; 3 Xinjiang Uygur Autonomous Region Academy of Animal Sciences, Urumqi, Xinjiang China

**Keywords:** Genome-environment association, Environmental adaptation, Frigid environments, Whole genome resequencing, Select signal analysis

## Abstract

Understanding the genetic mechanism of cold adaptation in cashmere goats and dairy goats is very important to improve their production performance. The purpose of this study was to comprehensively analyze the genetic basis of goat adaptation to cold environments, clarify the impact of environmental factors on genome diversity, and lay the foundation for breeding goat breeds to adapt to climate change. A total of 240 dairy goats were subjected to genome resequencing, and the whole genome sequencing data of 57 individuals from 6 published breeds were incorporated. By integrating multiple approaches such as phylogenetic analysis, population structure analysis, gene flow and population history exploration, selection signal analysis, and genome-environment association analysis, an in-depth investigation was carried out. Phylogenetic analysis unraveled the genetic relationships and differentiation patterns among dairy goats and other goat breeds. Through signal analysis (θπ, FST, XP-CLR), we identified numerous candidate genes associated with cold adaptation in dairy goats (*STRIP1, ALX3, HTR4, NTRK2, MRPL11, PELI3, DPP3, BBS1*) and cashmere goats (*MED12L, MARC2, MARC1, DSG3, C6H4orf22, CHD7, MYPN, KIAA0825, MITF*). Genome-environment association (GEA) analysis confirmed the link between these genes and environmental factors. Moreover, a detailed analysis of the critical genes *C6H4orf22* and *STRIP1* demonstrated their significant roles in the geographical variations of cold adaptation and allele frequency differences among different breeds. This study contributes to understanding the genetic basis of cold adaptation, providing crucial theoretical support for precision breeding programs aimed at improving production performance in cold regions by leveraging adaptive alleles, thereby ensuring sustainable animal husbandry.

**Supplementary Information:**

The online version contains supplementary material available at 10.1007/s44154-025-00254-5.

## Introduction

Due to the rapid changes in global climate, extreme weather is becoming increasingly serious each year (Coumou and Rahmstorf 2012). This situation has negatively impacted the development of animal husbandry, particularly in goats, which are vital sources of livestock products for humans (Tabassum-Abbasi et al. 2016). Consequently, human food security is at considerable risk (Godde et al. 2021; Lake et al. 2012; Lobell et al. 2011, p. 1). Studying the differences in the adaptability of goats in tropical and cold regions can significantly enhance the overall adaptability of the species, ultimately improving economic traits such as milk production (Amiri Ghanatsaman et al. 2023; Zhang et al. 2024). However, the genetic basis of this adaptability and how environmental factors shape genomic diversity remains poorly understood. Identifying the genes associated with environmental adaptation in goats from various climates is essential for comprehending the mechanisms of adaptation. Furthermore, it is crucial to develop livestock breeds that can thrive in changing climates, thereby supporting a growing global population.

The goat is one of the earliest domesticated animals, with a history that dates back about 10,000 years (Zheng et al. 2020). Wild goats, the ancestors of domesticated goats, are known for their strong adaptability and typically inhabit high-altitude, mountainous, or hilly regions. This adaptability allows them to withstand extreme weather conditions and highlights their ability to forage in environments with scarce resources (Amills et al. 2017; Benjelloun et al. 2024; Naderi et al. 2008; Ropiquet and Hassanin 2006). As humans have diversified their use of goats, these animals have been classified into different types based on their primary purpose: dairy, meat, and cashmere. Each type has specific environmental adaptability (Danso et al. 2024; MacHugh and Bradley 2001; Vijh et al. 2023). For example, cashmere goats are mainly found in arid and cold regions, where the climate supports their growth (Chang et al. 2024). Meat goats thrive in humid or warm areas, which are more conducive to fattening (Teixeira et al. 2024). Dairy goats are predominantly located in temperate and subtropical regions, where they provide high-quality protein products. Among these types, dairy goats and cashmere goats are particularly important due to their efficient production capacity (Lima et al. 2022; Zhao et al. 2024).

In the context of global climate change, research on the adaptability of goats has become increasingly urgent. Tropical and cold environments present different challenges to the physiological characteristics, growth, and reproductive abilities of goats (Danmaigoro et al. 2024; Habeeb et al. 2023). Therefore, understanding the genetic mechanisms that contribute to these differences is essential for improving the breeding of goats. This study aims to explore the genetic relationships among various goat breeds through the analysis of their population genetic structure, kinship relationships, gene flow, and demographic history across different environmental regions. Additionally, selection signal analysis will be conducted to identify genes that have been selected in different environments. Furthermore, landscape genomics and environmental association analyses will be employed to investigate the primary environmental factors influencing the adaptability of goat breeds, as well as the genetic markers linked to these factors. This research not only aims to establish a foundation for identifying and analyzing adaptive genes in goats but may also provide scientific support for enhancing production performance and ensuring global food security.

## Results

### Statistical analysis of sequencing data

**Fig. 1:**
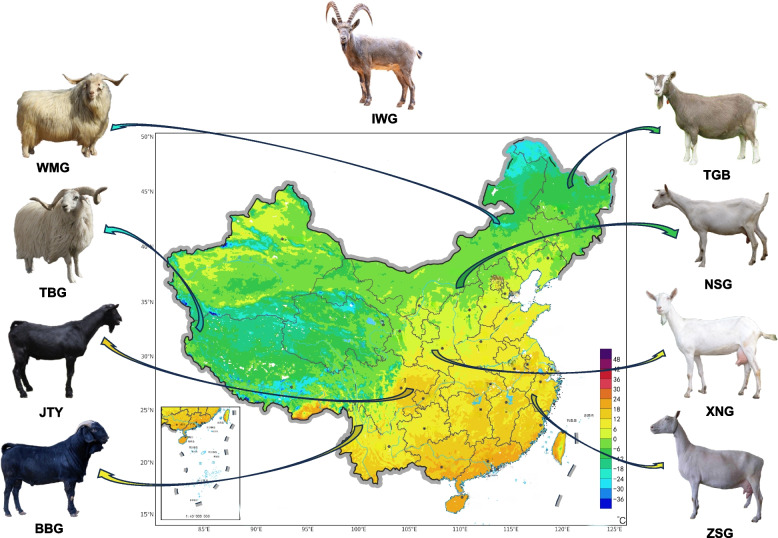
The geographic distribution of goat breeds. Xinong dairy Goats (XNG, *n* = 80) from Shaanxi Province, Saanen dairy goats collected from Inner Mongolia (NSG, *n* = 80), and Saanen dairy Goats from Zhejiang Province (ZSG, *n* = 80). Tugenburg dairy Goat (TGB, *n* = 10) from Heilongjiang Province, Yunnan Black Goat (BBG, *n* = 10) from Yunnan Province, Jintang Black Goat (JTY, *n* = 10) from Sichuan Province, Tibetan Goat (TBG, *n* = 9) from Xizang, Wuzhumuqin Goat (WMG, *n* = 9) from Inner Mongolia, Iranian Wild Goat (IWG, *n* = 9) from Iranian

A total of 240 dairy goats were selected for genome resequencing (Supplementary Table S1). These datasets were also analyzed together with the published whole-genome sequencing (WGS) datasets of 57 individuals from 6 breeds (Supplementary Tables S2). Among the 8 domestic goat breeds, Xinong Dairy goat (XNG), Tugenburg Dairy Goat (TGB), and Saanen Dairy Goat (NSG, ZSG) are the dairy goat breeds located in China; Yunnan Black Goat (BBG), Jintang Black Goat (JTY), Tibetan Goat (TBG), and Wuzhumuqin Goat (WMG) are nondairy goat breeds (Fig. 1). The remaining goat breeds include Iranian wild goat (IWG).

A total of 6.84 TB paired-end DNA sequence data were obtained from 297 goats. All goats were sequenced with an average 13.62-fold depth of the genome (4.3∼31.8 ×) and an average genome coverage of 97.49% (Supplementary Tables S1–S2). Of the reads, 99.60% were mapped to the latest goat reference genome ARS1.2 (GCA_001704415.2) (Supplementary Tables S1–S2). We detected 39,275,107 high-quality single-nucleotide polymorphisms (SNPs), with 1.17% (836,600) located in exonic regions (Supplementary Table S3). The average transition-to-transversion (Ti/Tv) ratio was 2.38 for all goat samples, which indicated relatively low potential random sequencing errors (Supplementary Table S4).

### Population structure and phylogenetic relationships

**Fig. 2:**
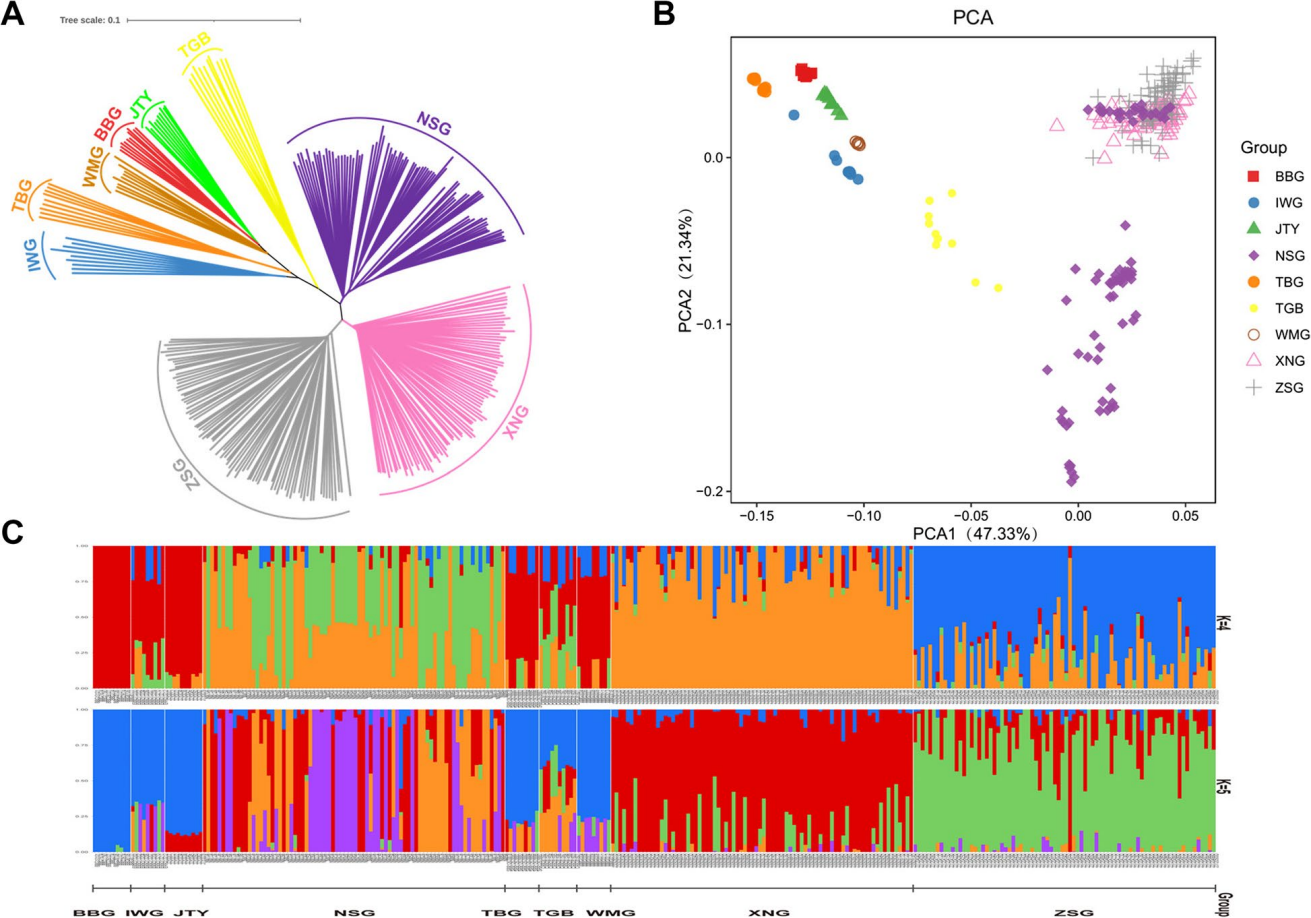
The structures of goats from 9 populations. **A** Phylogenetic tree. Phylogenetic relationships were estimated using the neighbor-joining method. **B** PCA. Principal components 1 (47.33%) and 2 (21.34%) for the goats. **C** Genetic structure of goats using ADMIXTURE when K ranged from 4 to 5

To infer genetic and evolutionary relationships of goat breeds adapted to diverse temperature environments, we generated high-quality whole genome sequencing data of 297 individuals from 9 populations, as previously mentioned. Next, a phylogenetic tree from a total of 9 goat breeds with 297 goats was constructed using the neighbor-joining (NJ) algorithm according to pairwise genetic distances between populations according to the polymorphic SNPs in the whole genome. The tree revealed that individuals from the same geographic region tended to cluster into a single group (Fig. 2A). IWG, as the ancestor of the domestic goat, was in the root of the tree and clustered together with TBG, WMG, BBG, and JTY, which TBG is an ancient native breed raised in Tibetan Plateau, TBG is closer to IWG. Those 4 breeds first branched off, then TBG, WMG, BBG, and JTY successively branched off in order of their geographic position from north to south. Dairy goats branched off relatively late as they were a breed selected by artificial cultivation at a later stage, and dairy goats from different geographical locations gathered together. These findings were corroborated by both principal component analysis (PCA) and population structure analysis (Fig. 2B and C). We further performed genetic structure analyses to partition all goats into groups by varying the number of presumed ancestral populations (K = 2–7, Fig. 2C, supplementary Fig. S1). When K = 4, we observed 4 separate clusters: NSG, XNG, and ZSG are three dairy goat breeds, and the rest are wild goat (IWG) and local goat breeds (TBG, BBG, JTY, WMG).

### Migration events and population history

**Fig. 3:**
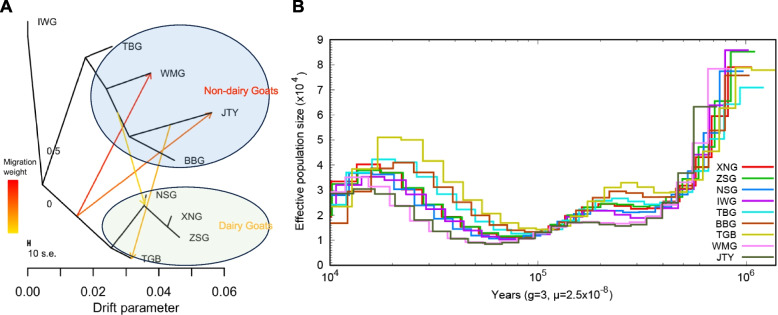
Demographic history of the goat population. **A** Pattern of population splits and mixtures among the nine populations. The drift parameter is proportional to Ne generations, where Ne is the effective population size. Scale bar shows ten times the average standard error of the estimated entries in the sample covariance matrix. Arrows indicate migration events, and a spectrum of heat colors indicate the migration weights of the migration events. **B** Estimated effective population size of the goats individuals with PSMC analysis based on SNP distribution

We then rebuilt the maximum likelihood tree and residual matrix of these breeds and added sequential migration events to the tree by using TreeMix, to address population history relationships and to deduce migration patterns among those different breeds (Fig. 3A, supplementary Fig. S2, S3). We found that one supposed migration edge produced a tree with the smallest residuals and consequently best fit the data. This edge provides evidence for the gene flow from IWG to the breeds in Northern China. In recent years, studies have provided a perspective on the pattern of gene flow between southern and Northern breeds in China, though no direct evidence has been presented to demonstrate that IWG has been crossbred with Northern breeds. Similar to the previous TreeMix finding, our data found gene flow between IWG, WMG, and JTY. The current study revealed gene flow between JTY and TGB, WMG, and NSG, which might be the use of different varieties for hybrid cultivation. These findings indicate that the differentiation time of dairy goats is different from that of non-dairy goats, with dairy goats being a high-intensity artificially selected breed that differentiates later. breeds and frequent gene flow among them, further implying the multiple and close relationships of these breeds.

To explore the demographic history of the 9 goat populations, the historical effective population sizes (Ne) of the goat ancestors were estimated using the PSMC method according to SNPs in the whole genome and the linkage disequilibrium among them. The results revealed that individuals from the same populations or the same geographic regions exhibited a highly similar pattern of demographic trajectories (Fig. 3B). The Ne of the 9 populations fluctuated over time with the earth’s climate (In the time of the Quaternary ice ages and the Last Interglacial until the Last Glacial Maximum), with two expansions and two bottlenecks in the demographic history.

### Signal analysis reveals goat’s cold adaptability

**Fig. 4:**
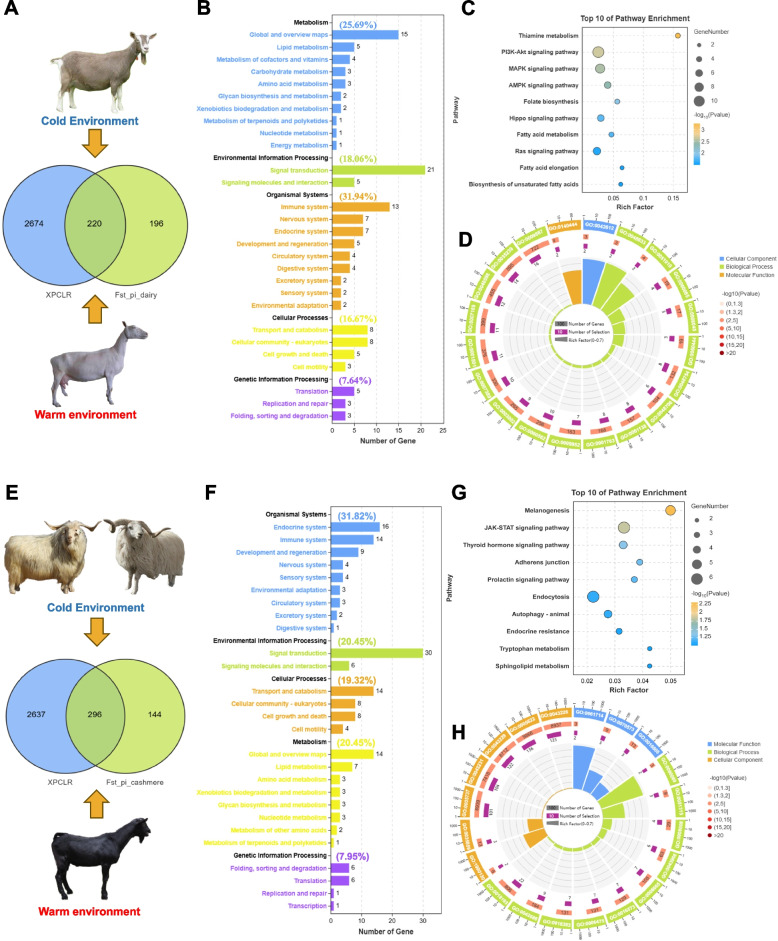
Selective sweep analysis of dairy goats and cashmere goats. **A** Venn diagram showing the gene overlaps among FST_Pi and XP-CLR for TGB with ZSG. **B** Distribution of gene quantity in different biological systems and cellular processes based on genes across significant selected regions from dairy goats. **C** KEGG pathway enrichment analysis based on genes across significant selected regions from dairy goats. **D** GO enrichment analysis based on genes across significant selected regions from dairy goats. **E** Venn diagram showing the gene overlaps among FST_Pi and XP-CLR for WMG, TBG with JTY. **F** Distribution of gene quantity in different biological systems and cellular processes based on genes across significant selected regions from cashmere goats. **G** KEGG pathway enrichment analysis based on genes across significant selected regions from cashmere goats. **H** GO enrichment analysis based on genes across significant selected regions from cashmere goats

In order to identify the selected areas for the adaptation of dairy goats to cold environments. We chose to conduct selection signal analysis on Toggenburg dairy goats living in cold environments and Saanen dairy goats living in warm environments (Fig. 4A). we compared the genomes of dairy goats by calculating the ratio of nucleotide diversity (θπ TGB/ZSG) and pairwise genetic differentiation (FST) with a 50 kb sliding window and a 25 kb sliding step. The top 5% threshold outlier windows of the two methods were combined, which was considered to be the specific region of dairy goats under positive selection (supplementary Fig. S4). In addition, we also conducted XP-CLR analysis, combining XP-CLR analysis with FST_PI The overlapping region is considered a candidate region, and 220 candidate genes were identified for functional enrichment in this region (Fig. 4A, Supplementary Table S5, S6, S7). This suggests that this region may be associated with the breed specificity of dairy goats. The functional enrichment analysis of 220 selected candidate genes showed that the KEGG pathway was divided into metabolism, genetic information processing, environmental information processing, cellular processes, and organismal systems (Fig. 4B). The proportion of organismal systems is the highest (31.94%), followed by metabolic systems (25.69%). The KEGG pathway is mainly enriched in signaling pathways such as Thiamine metabolism, Pl3K-Akt, MAPK, etc. (Fig. 4C, Supplementary Table S8). GO analysis found that these genes represent a series of biological processes, including cytokeleton nuclear membrane anchor activity (GO: 0144044, *P* = 0.0005), MHC class I protein complex (GO: 0042612, *P* = 0.0002), positive regulation of skeletal muscle tissue growth (GO: 0048633, *P* = 0.0002), etc. (Fig. 4D, Supplementary Table S9).

In order to identify the selected areas for the adaptation of cashmere goats to cold environments. We chose to conduct selection signal analysis on Tibetan goats, Wuzhumuqin cashmere goats long-term living in cold environments, and jintang black goats living in warm environments (Fig. 4E). we also combined XP-CLR analysis with FST_PI The overlapping region is considered a candidate region, and 296 candidate genes were identified for functional enrichment in this region (Fig. 4E, Supplementary Table S10, S11, S12). This suggests that this region may be associated with the breed specificity of cashmere goats. The functional enrichment analysis of 296 selected candidate genes showed that the KEGG pathway was divided into metabolism, genetic information processing, environmental information processing, cellular processes, and organismal systems (Fig. 4F). The proportion of organismal systems is the highest (31.82%), followed by metabolism (20.45%) and Environmental Information Processing (20.45%). The KEGG pathway is mainly enriched in signaling pathways such as melanogenesis, JAK-ATAT, Thyroid hormone signaling pathway, etc. (Fig. 4G, Supplementary Table S13). GO analysis found that these genes represent a series of biological processes, including folic acid receptor activity (GO:0061714, *P* = 0.0004), regulation of ribosome biogenesis (GO:0090069, *P* = 0.0005), GATOR1 complex (GO:1,990,130, *P* = 0.0019), etc. (Fig. 4H, Supplementary Table S14). The above results indicate that environmental adaptability involves complex physiological processes, and dairy goats and cashmere goats employ distinct physiological pathways for cold adaptation.

### Landscape genome and environmental association analysis

**Fig. 5:**
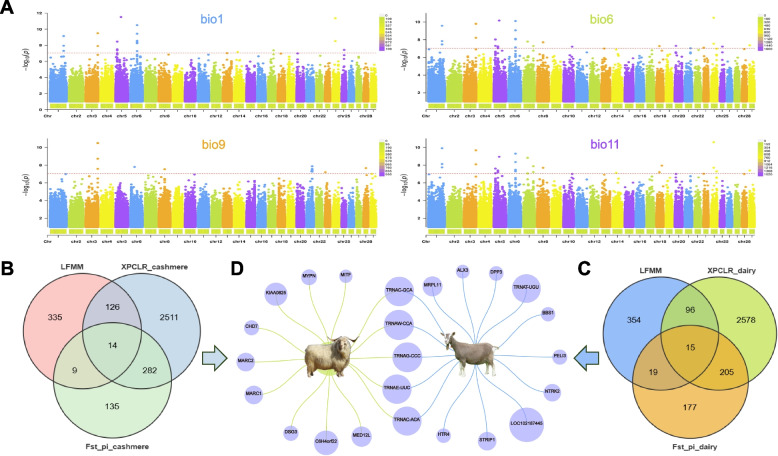
GEA analysis and Landscape genome in dairy goats and cashmere goats. **A** Manhattan plots showing candidate sites associated with the bio1, bio6, bio9, and bio11 related to environmental temperature in goats. Each dot represents the SNP for a given sliding window along the genome. Colors differentiate consecutive chromosomes. **B** Venn diagram showing the gene overlaps among FST_Pi, LFMM, and XP-CLR for WMG, TBG with JTY. **C** Venn diagram showing the gene overlaps among FST_Pi, LFMM, and XP-CLR for TGB with ZSG. **D** Environment-related genes shared by and specific to dairy goats and cashmere goats

To explore key genetic loci closely associated with environmental factors. We used population genomics and climatic data to investigate the genetic adaptation to climate change in goats by combining two genotype-environment association (GEA) methods and three selection detection methods. GEA based on RDA and LFMM was used to identify putative climate adaptive SNPs. RDA, a multivariate linear regression method, was used to identify the weak multilocus signatures of selection. A set of genomewide LD-pruned SNPs was used as response variables in the RDA, while four temperature-associated environmental factors (BIO1, BIO6, BIO9, BIO11) were used as explanatory variables after filtering to avoid multicollinearity. The results indicated that the overall model has a very high level of significance, and The first four axes of RDA can explain the variance in the RDA model and. (supplementary Fig. S6). SNP loading calculated in the RDA showed a normal-like distribution (supplementary Fig. S7), in which the two ends of the distribution show the highest relationship with the environmental predictors. We then conducted an association analysis between BIO1, BIO6, BIO9, BIO11, and genome-wide SNPs using LFMM (Fig. 5A). Through screening of SNPs and candidate genes associated with environmental variables based on q-values calculated by false discovery rate (FDR) correction, we identified a total of 484 candidate genes that were significantly associated with BIO1, BIO6, BIO9, and BIO11 (Supplementary Table S15). Afterward, the genes identified by the selection signal analysis of cashmere goats were combined with the genes identified by LFMM for joint analysis (Fig. 5B), and a total of 14 genes associated with the environment were identified (*MED12L, TRNAC-GCA, MARC2, MARC1, TRNAG-CCC, DSG3, TRNAE-UUC, C6H4orf22, TRNAC-ACA, TRNAW-CCA, CHD7, MYPN, KIAA0825, MITF*). Fifteen genes associated with the environment were identified through joint analysis of genes identified by signal analysis and LFMM in dairy goats (Fig. 5C) (*TRNAC-GCA, TRNAG-CCC, STRIP1, ALX3, LOC102187445, TRNAE-UUC, HTR4, TRNAC-ACA, TRNAW-CCA, NTRK2, TRNAT-UGU, MRPL11, PELI3, DPP3, BBS1*). The cold adaptation genes shared by both cashmere goats and dairy goats are small molecule RNAs such as tRNA, which play a crucial role in protein synthesis. Other genes play an important role in cold resistance in different goat breeds (Fig. 5D).

### Adaptive genetic variation under clod environments in goats

**Fig. 6:**
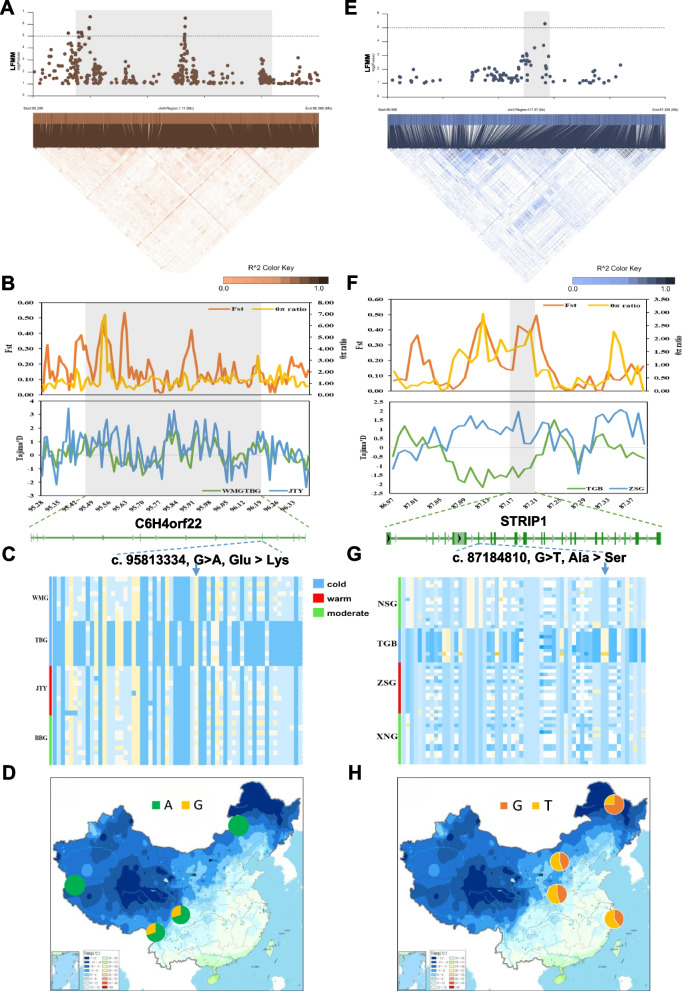
Candidate adaptive genes analysis. *C6H4orf22* (**A**–**D**) and *STRIP1* (**E**–**H**). **A**,**E** Manhattan plots and LDblock plots showing candidate genes (*C6H4orf22* and *STRIP1*) associated with environmental temperature in dairy goats and cashmere goats. **B**,**F** Fst, θπ ratio, and Tajima’D values are selected genes in dairy and cashmere goats. The green pattern diagram is the structural diagram of genes. **C**, **G** Gene haplotype heatmap of selected genes in goats living at different environmental temperatures. **D**, **H** The allele frequency distribution of the selected gene in different geographical locations

In order to conduct a more detailed analysis of genes related to cold resistance in goats. We conducted a comprehensive analysis of genes on chromosomes chr1, chr3, and chr6 (*MED12L, STRIP1, ALX3, C6H4orf22*) with significant peaks in LFMM analysis, as well as *MITF* related to environmental adaptability. We found the *C6H4orf22* located at chr6 in cashmere goats. The *C6H4orf22* has significant loci and certain LDblock blocks in LFMM (Fig. 6A). Through Fst and Tajima’D analysis, it was found that the *C6H4orf22* was strongly selected in cashmere goats (Fig. 6B). And a non-synonymous mutation (c. 95,813,334, G > A, Glu > Lys) was found in the *C6H4orf22*, which exhibits different patterns in cold and warm environments (Fig. 6C). Furthermore, by analyzing the allele frequency distribution of SNP loci, it was found that the allele frequency distribution varies with the geographical environment from north to south (Fig. 6D). In addition, we discovered the *STRIP1* located at chr3 in dairy goats, which has significant loci in LFMM and certain LDblock blocks (Fig. 6E). Through Fst and Tajima’D analysis, it was found that the *STRIP1* is strongly selected in TGB (Fig. 6F). And a non-synonymous mutation (c. 87,184,810, G > T, Ala > Ser) was found in the *STRIP1*, which exhibited different patterns in cold and warm environments (Fig. 6G). Furthermore, by analyzing the allele frequency distribution of SNP loci, it was found that the allele frequency distribution varies with the geographical environment from north to south (Fig. 6H). The *MED12L, ALX3,* and *MITF* also exhibit similar patterns to the *C6H4orf22* and *STRIP1*, but no non-synonymous mutations were found (Supplementary Fig. S8, S9, S10).

## Discussion

The temperature fluctuations brought about by global climate change pose significant challenges to the adaptability of livestock, especially for those breeds that have undergone long-term breeding under specific climate conditions (Thornton et al. 2014, 2009). In this study, we conducted whole-genome resequencing on 297 goats from different geographical regions, aiming to explore the genetic mechanisms of environmental adaptability, especially the differences in adaptability in cold regions. Our research results show that different breeds of goats exhibit significant diversity at the genomic level, which is closely related to their geographical environment. Phylogenetic analysis indicates that goat individuals within the same geographic region tend to cluster together, indicating that geographic isolation plays a crucial role in the differentiation of goat breeds. In addition, we also detected multiple gene flow events, which further shaped the genetic structure of goat breeds, indicating that there was extensive gene exchange between different breeds in the evolution of goats, reflecting their complex and close kinship relationships. Critically, the observed genetic patterns reflect the synergistic effects of three evolutionary forces: (1) Geographic isolation promoted population divergence; (2) Breed management practices facilitated adaptive introgression; and (3) Artificial selection intensified allele frequency shifts in genes underlying production traits. This integrative framework aligns with findings in reindeer (Lin et al. 2019) and yak (Qiu et al. 2012), though goats exhibit unique trade-offs between environmental adaptation and human-selected productivity traits.

By selecting signal analysis, we successfully identified multiple genes and pathways related to goat cold adaptation. In the comparison of Toggenburg dairy goats and Saanen dairy goats, we found that the selected genes were mainly enriched in biological systems and metabolic pathways, such as thiamine metabolism, PI3K-Akt, and MAPK signaling pathways, which play important roles in physiological processes such as heat production (Arianti et al. 2023), energy metabolism (“Eicosapentaenoic acid-mediated activation of PGAM2 regulates skeletal muscle growth and development via the PI3K/AKT pathway,” 2024; Manzetti et al. 2014; Wang et al. 2024), and immune regulation (Liu et al. 2007). In the analysis of Tibetan goats, Wuzhumuqin cashmere goats, and Jintang black goats, the identified candidate genes mainly involve melanin production, JAK-STAT, and thyroid hormone signaling pathways, which are closely related to temperature regulation (Roulin 2014; Slominski et al. 2004), hair development (Harel et al. 2015; He et al. 2022), heat production (Iwen et al. 2018; Silva 2006), and metabolic regulation (Sinha and Yen 2024). These results indicate that dairy goats and cashmere goats adopt different physiological strategies when adapting to cold environments, which may be related to their respective production performance and ecological roles.

Landscape genomics and environmental association analysis further reveal the close relationship between environmental factors and genetic variation. We identified multiple candidate genes related to temperature using the GEA method, which have different functions in different goat breeds, together playing a role in resisting the cold (Supplementary Fig. S11). While tRNA genes showed significant genotype-environment associations, their role in cold adaptation is likely indirect. Changes in tRNA abundance or modifications (Guo et al. 2023) may optimize translation efficiency under metabolic stress in cold environments—a phenomenon observed in hibernating mammals. In addition, we also found genes such as *MED12L, MARC2, MARC1, DSG3, C6H4orf22, CHD7, MYPN, KIAA0825, and MITF*. showed environmental correlations in cashmere goats. *MED12L, KIAA0825,* and *CHD7* may be related to villous growth and energy metabolism (Gaitskell-Phillips et al. 2022; Ross et al. 2017; Zhou et al. 2021). *MARC2* and *MARC1* may be related to mitochondrial metabolism (Jones et al. 2024). *DSG3* may be related to villous traits and high-altitude adaptability (Guo et al. 2019). *MYPN* regulates muscle contraction to resist cold (Filomena et al. 2020). The previously reported *MITF* gene is associated with coat color and environmental adaptability in sheep (Hu et al. 2019). Furthermore, we also identified a series of genes related to temperature in dairy goats, including *STRIP1, ALX3, HTR4, NTRK2, MRPL11, PELI3, DPP3* and *BBS1*. *ALX3* is related to fiber color (Mendoza et al. 2019). *DPP3* and *HTR4* may be related to the cardiovascular system and lungs (Korytina et al. 2017; Ye et al. 2022). *NTRK2* is associated with sheep reproductive traits (Yang et al. 2024). *PELI3* and *BBS1* may be related to signal transduction function (Hu and Sun 2016; Masek et al. 2022). *MRPL11* may be related to mitochondrial function (Ma et al. 2020). It is worth noting that the *C6H4orf22* and *STRIP1* genes exhibit strong selection signals and correlations in cashmere goats and dairy goats, respectively. *C6H4orf22* is associated with environmental adaptability in sheep, as well as with reproductive traits and wool traits in sheep (Niu et al. 2024; Zhao et al. 2021). However, *STRIP1* plays an important role in glucose and lipid metabolism(Hwang and Pallas 2014), *STRIP1* also is involved in *Stk25* gene product function related to expression of uncoupling protein 3, which is located primarily to Brown adipose tissue (BAT) and muscle (Azzu et al. 2010; Pravenec et al. 2018). Brown adipose tissue is an important thermogenic organ in small mammals and newborns (Villarroya et al. 2017), producing heat through non shivering thermogenesis to help maintain body temperature, especially in cold environments where it is crucial for maintaining temperature stability (Klingenspor 2003; Smith 1964). Muscle tissue plays a crucial role in the process of shivering induced heat production, generating heat through muscle contraction (Periasamy et al. 2017; Rowland et al. 2015). Further analysis of the *C6H4orf22* and *STRIP1* genes revealed significant differences in allele frequency distribution across different geographical environments, indicating that these genes may be an important genetic basis for goats to adapt to cold environments.

This study provides important insights into the genetic mechanisms of environmental adaptation in goats. Our research results not only help identify key genes and pathways related to adaptability but also provide valuable genetic resources for the breeding of future goat breeds. In the context of global climate change, these findings have important practical significance for improving the production performance of goats and enhancing their adaptability to environmental changes. They are expected to provide strong support for ensuring global food security. However, our research also has certain limitations, such as a relatively small sample size that may not cover the genetic diversity of all goat breeds. Future research can further expand the sample size and delve into the impact of other environmental factors on goat adaptability, in order to more comprehensively reveal the genetic mechanisms of goat adaptability.

## Conclusion

In conclusion, the present study provided a comprehensive analysis of the genetic adaptation of dairy and cashmere goats to different environments. The analysis included 297 individuals from 9 breeds, covering both warm and cold regions. Patterns of genetic diversity and population structure revealed the evolutionary relationships and gene flow among these breeds. Through selection signal analysis, candidate genes associated with cold adaptation were identified in dairy and cashmere goats, with different physiological pathways involved in each. Landscape genomics and environmental association analysis further pinpointed key genetic loci and genes related to environmental factors. tRNA was found to be shared in both types of goats, while other genes like *C6H4orf22* and *STRIP1* in cashmere and dairy goats showed strong selection and specific mutations related to cold adaptation, with allele frequencies varying geographically. Overall, these results suggest that the genetic differences in these goat populations are crucial for their adaptation to diverse environments. These results contribute to a deeper understanding of the adaptive genetic mechanisms in goats, the genomic data obtained in this research also will be valuable for the breeding of dairy and cashmere goats to enhance their adaptability in the face of climate change.

## Materials and methods

### Ethics statement

The study received approval from the Institutional Animal Care and Use Committee at Northwest A&F University. All relevant institutional and national guidelines regarding the care and welfare of animals were strictly followed throughout the sampling procedures.

### Sample collection and sequencing

A total of 297 samples were included in this study, comprising Saanen dairy goats from three regions: Shaanxi Province (XNG, 34.12°N, 108.07°E; n = 80), Inner Mongolia (NSG, 40.35°N, 112.07°E; n = 80), and Zhejiang Province (ZSG, 29.47°N, 118.54°E; n = 80). For each goat, 5 mL of blood was collected from the jugular vein, and DNA extraction was performed using the standard phenol–chloroform method. The samples with determined DNA concentrations were subjected to whole-genome sequencing by Huada Company, utilizing paired-end libraries constructed with the Huada T7 platform. Each individual had an average insert size of 500 bp and an average read length of 150 bp. Additionally, resequencing data from 57 individuals were downloaded from public databases. This included six different breeds: Tugenburg Dairy Goat (TGB, *n* = 10), Yunnan Black Goat (BBG, *n* = 10), Jintang Black Goat (JTY, *n* = 10), Tibetan Goat (TBG, *n* = 9), Wuzhumuqin Goat (WMG, *n* = 9), and Iranian Wild Goat (IWG, *n* = 9).

### Read mapping and variant calling

The study utilized Trimmomatic v0.39 (Bolger et al. 2014) for filtering the paired-end sequences. Next, BWA-MEM (v0.7.15-r1140) (Li and Durbin 2009) aligned the clean data with the goat reference genome ARS1.2 (GCF_001704415.2, https://www.ncbi.nlm.nih.gov/datasets/genome/GCF_001704415.2/). Subsequently, samtools (Li et al. 2009) was utilized to construct a BAM file index for mapping. The BAM files were then sorted and potential duplicate reads were removed utilizing the Picard (http://broadinstitute.github.io/picard) tool. After mapping, SNP calling was performed using the “Haplotype Caller,” “Genotype GVCFs,” and “Select Variants” modules in the GATK genomic analysis tool package (GATK, version 3.8–1-0-gf15c1c3ef) (McKenna et al. 2010). The initial SNPs were filtered with the “Variant Filtration” module based on the parameters: “QD < 2.0, FS > 60.0, MQ < 40.0, MQRankSum < − 12.5, ReadPosRankSum < − 8.0, and SOR > 3.0,” and an average sequencing depth of variants within the range “ < 1/3 × and > 3 × ” for all individuals. Lastly, the ANNOVAR (Wang et al. 2010) software was utilized for functional annotation of the SNPs.

### Population genomic analysis

Three methods are used for VCF filtering and population structure estimation: (a) constructing a neighbor-joining tree with MEGA v10.2.6 (Kumar et al. 2018) and visualizing it using iTOL (Letunic and Bork 2021); (b) principal component analysis (PCA) with EIGENSOFT v5.0 (Patterson et al. 2006) software; and (c) conducting population structure analysis using ADMIXTURE v1.3.0 (Alexander and Lange 2011). Cross-validation is employed to calculate the cross-validation error and determine the optimal K value (assuming the ancestral population K value ranges from 2 to 8). VCFtools (Danecek et al. 2011) are used to analyze nucleotide diversity for each breed in non-overlapping 50 kb windows. Next, the PopLDdecay (Zhang et al. 2019) software is used to calculate the decay of LD to assess the physical distance between SNPs within haplotype blocks of different breeds.

### Demographic history and potential distribution prediction

PSMC (v0.6.5) (Li and Durbin 2011) was used to infer the history of effective population size (Ne) of P. cathayana (μ = 2.5 × 10⁻⁸, g = 3 years). Fastsimcoal2 (v2.1) (Excoffier et al. 2013) was employed to derive the best-fitting demographic model, estimate the population differentiation time, and determine the per-generation migration rate of gene flow between clades. TreeMix (v1.11) (Pickrell and Pritchard 2012) was utilized to model gene flow among subgroups of P. cathayana. This analysis inferred the maximum likelihood tree and identified potential gene flow based on the residual covariance matrix. Six migration event models were established, and the optimal model was selected by considering the AIC value and stability.

### Detection of selective sweeps

Based on the characteristics and origins of the goat breeds, Divide goats into different environmental levels. We then compared the genomes of these goat breeds and estimated the signal scanning regions using a combination of nucleotide diversity (θπ) and fixation index (FST) with VCFtools, employing 50 kb sliding windows and 25 kb sliding steps. Additionally, Tajima’s D(Korneliussen et al. 2013) statistic and cross-population composite likelihood ratio test (XP-CLR) (Chen et al. 2010) were employed to identify potential regional differences between different breeds. XP-CLR is a likelihood method for detecting selective sweeps by jointly modeling multi-locus allele frequency differences between two groups. The scanned regions detected from the intersection of the two parameters with the highest 5% threshold were annotated to identify candidate genes. Lastly, Bedtools (Quinlan and Hall 2010) was used to annotate the selected regions for subsequent analysis.

### Collection of bioclimatic variable data

To evaluate the impact of environmental factors, we obtained data from WorldClim that included 19 bioclimatic variables (Bio1–19) covering the period from 1970 to 2000, with a resolution of 5 arcminutes. We extracted these variables using DIVA-GIS (v10.8). We calculated the Pearson correlation between the environmental variables and ultimately retained four variables for further analysis.

### Genotype–environment association analysis

The Latent Factor Mixed Model (LFMM) was utilized to investigate the relationship between outlier SNPs and four environmental variables, aiming to identify significant Latent Environmental Associations (EAL) (Caye et al. 2019). LFMMs were implemented using the R package for Latent Environmental Association analysis, incorporating natural genetic structure as a random effect. SNP loci with |z| values ≥ 4 and P-values ≤ (0.05/Ne) associated with at least one environmental variable were categorized as key EALs (Frichot et al. 2013). To better understand the independent effects of environmental variables on adaptability, a partial Redundancy Analysis (RDA) model was conducted. The key adaptive EALs served as response variables, while four environmental variables acted as explanatory variables. Furthermore, geographical factors were controlled for by including longitude and latitude as additional variables (Rellstab et al. 2015). The RDA function from the R package vegan was employed to carry out the partial RDA analysis, and the anova.cca function was used to assess the significance of the environmental variables. The proportion of genetic variation explained by each RDA axis was calculated. SNPs that fell on the tails of the distribution in RDA axes 1–3, with a 95% confidence interval as the cutoff, were regarded as candidate loci and subsequently underwent genomic annotation (Hoffmann et al. 2021).

### Enrichment analyses and visualization

The enrichment module in KOBAS (Bu et al. 2021) was utilized to identify pathways from the Kyoto Encyclopedia of Genes and Genomes (KEGG) as well as Gene Ontology (GO) terms that showed statistically significant associations (at a significance level of *p* ≤ 0.05). This comprehensive analysis allowed for a detailed exploration of both the biological functions and the molecular pathways potentially involved. Subsequently, the results were visualized through the OmicShare tool, enabling a more intuitive understanding and interpretation of the complex data obtained from the enrichment analysis.

## Supplementary Information

Supplementary Material 1.

Supplementary Material 2.

## Data Availability

This work was supported by the Genome Sequence Archiv in National Genomics Data Center, China National Center for Bioinformation/Beijing Institute of Genomics (CRA017705). Other data that supported the discovers could been obtained from the corresponding authors.

## Abbreviations

WGS: Whole-genome sequencing
XNG: Xinong dairy goat
TGB: Tugenburg dairy Goat
BBG: Yunnan Black Goat
JTY: Jintang Black Goat
TBG: Tibetan Goat
WMG: Wuzhumuqin Goat
IWG: Iranian wild goat
Ti/Tv: Transition-to-transversion
NJ: Neighbor-joining
PCA: Principal component analysis
PSMC: Pairwise Sequentially Markovian Coalescent
GEA: Genome-environment association
RDA: Redundancy Analysis
LFMM: Latent Factor Mixed Model
KEGG: Kyoto Encyclopedia of Genes and Genomes
GO: Gene Ontology
