# Supplementary material for: Landscape genomics analysis reveals the genetic basis underlying cashmere goats and dairy goats adaptation to frigid environments
Source: Stress Biol. 2025 Sep 9;5(1):56. doi: 10.1007/s44154-025-00254-5 (PMC12420540; doi:10.1007/s44154-025-00254-5)
Supplement: Supplementary file 2 — Supplementary Material 2. [file 44154_2025_254_MOESM2_ESM.docx]

**supplemental figure**


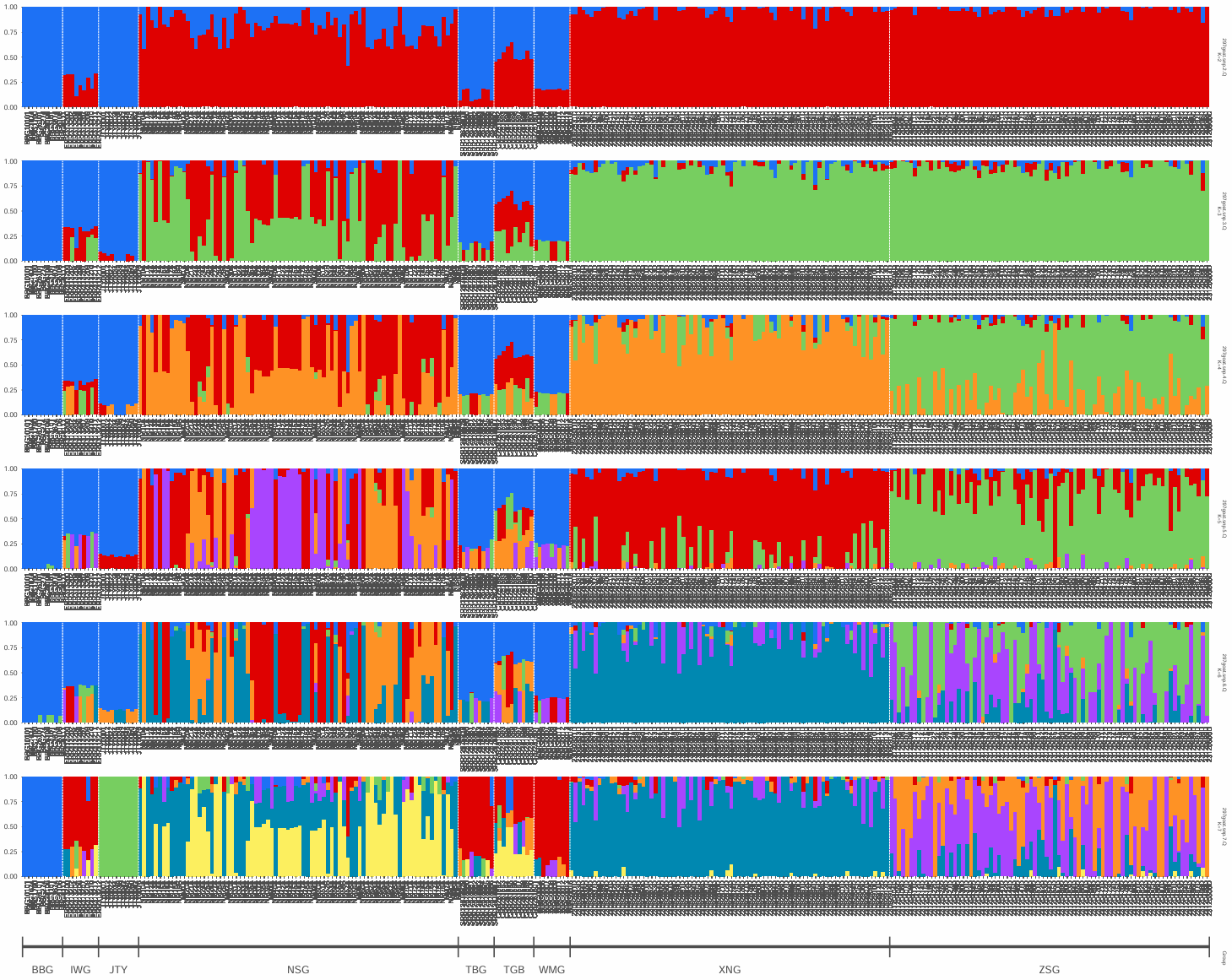


**Figure S1** Genetic structure of cattle using ADMIXTURE when K ranged from 2 to 7.


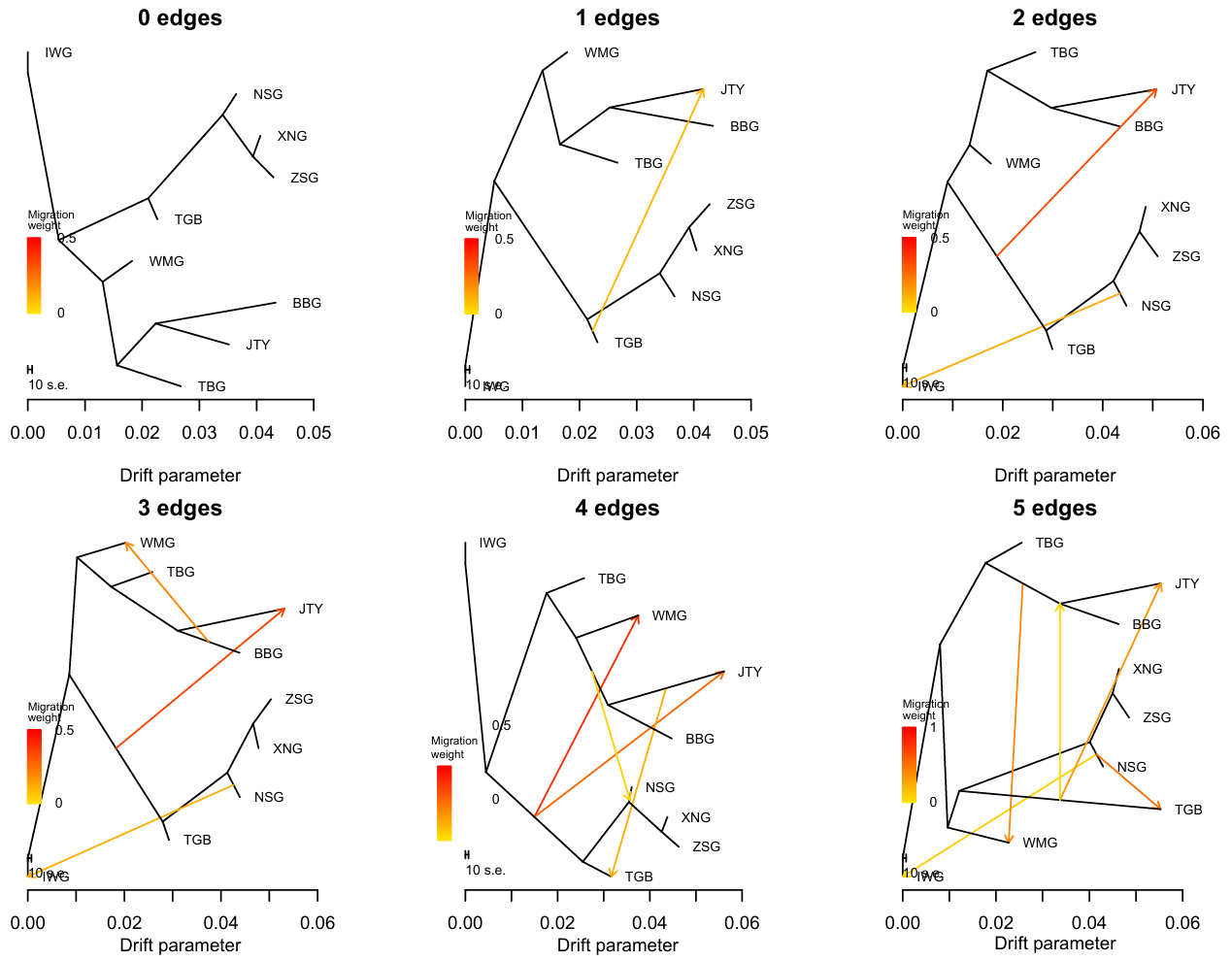


**Figure S2** The maximum likelihood tree of our 8 breeds and one “outgroup” breeds, and the likelihood tree assuming zero to five migration events using TreeMix. Arrows indicate migration events, and a spectrum of heat colors indicate the migration weights of the migration events.


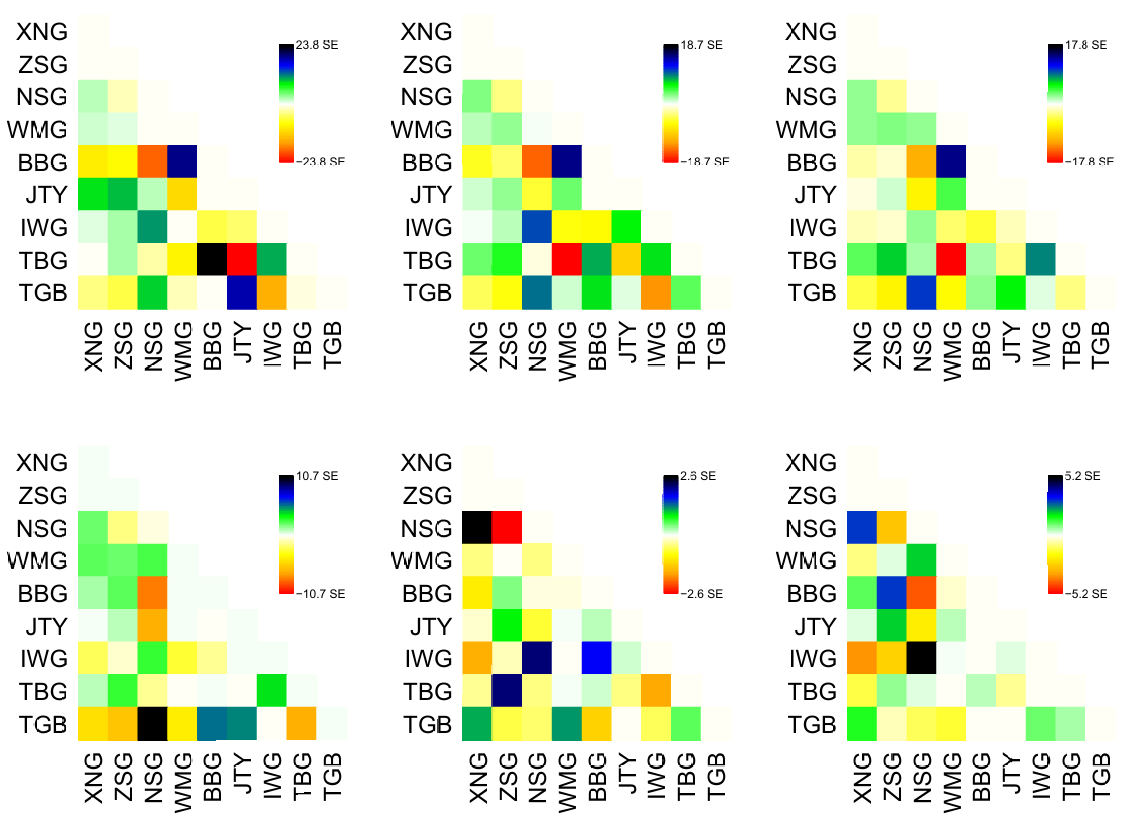


**Figure S3** The maximum likelihood tree of our 8 breeds and one “outgroup” breeds, and the the residual matrix of the maximum likelihood tree assuming zero to five migration events using TreeMix. The scale bar represents ten times the average standard error (s.e.) of the values in the covariance matrix. Positive residuals indicate pairs of breeds where thefit might be improved by adding additional edges.


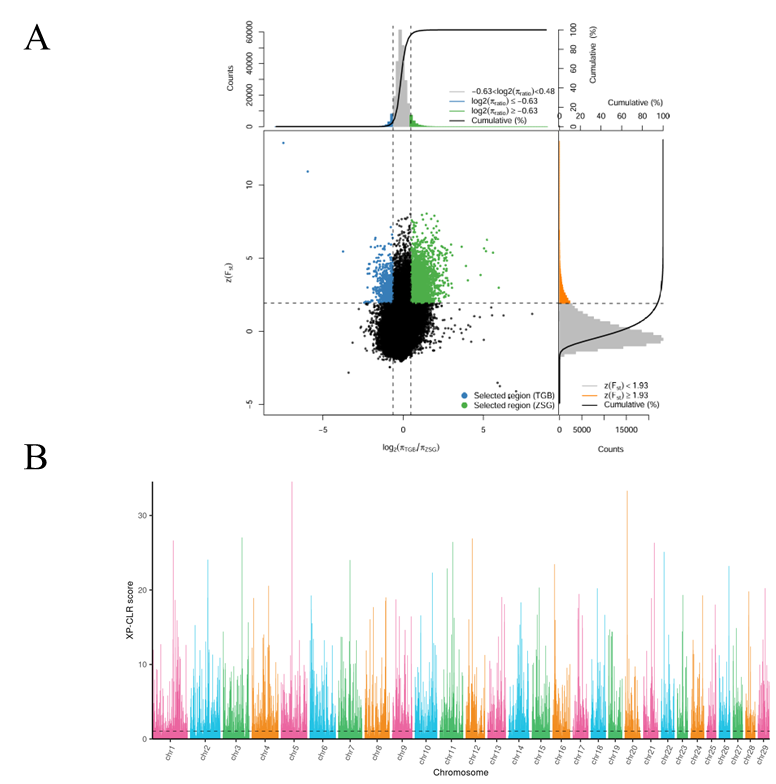


**Figure S4** Selective sweep analysis of dairy goats. (A) Distribution of log2 (θπ ratios) and FST values calculated in 50-kb sliding windows for genomic SNPs in dairy goats. (B) Distribution of XP-CLR calculated in 50-kb sliding windows for genomic SNPs in dairy goats.


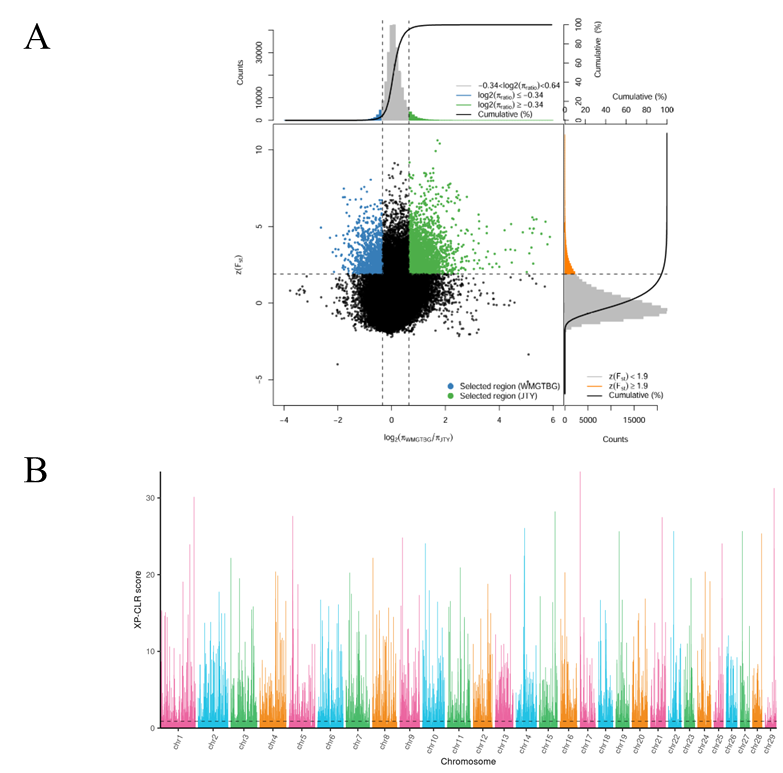


**Figure S5** Selective sweep analysis of cashmere goats. (A) Distribution of log2 (θπ ratios) and FST values calculated in 50-kb sliding windows for genomic SNPs in cashmere goats. (B) Distribution of XP-CLR calculated in 50-kb sliding windows for genomic SNPs in cashmere goats.


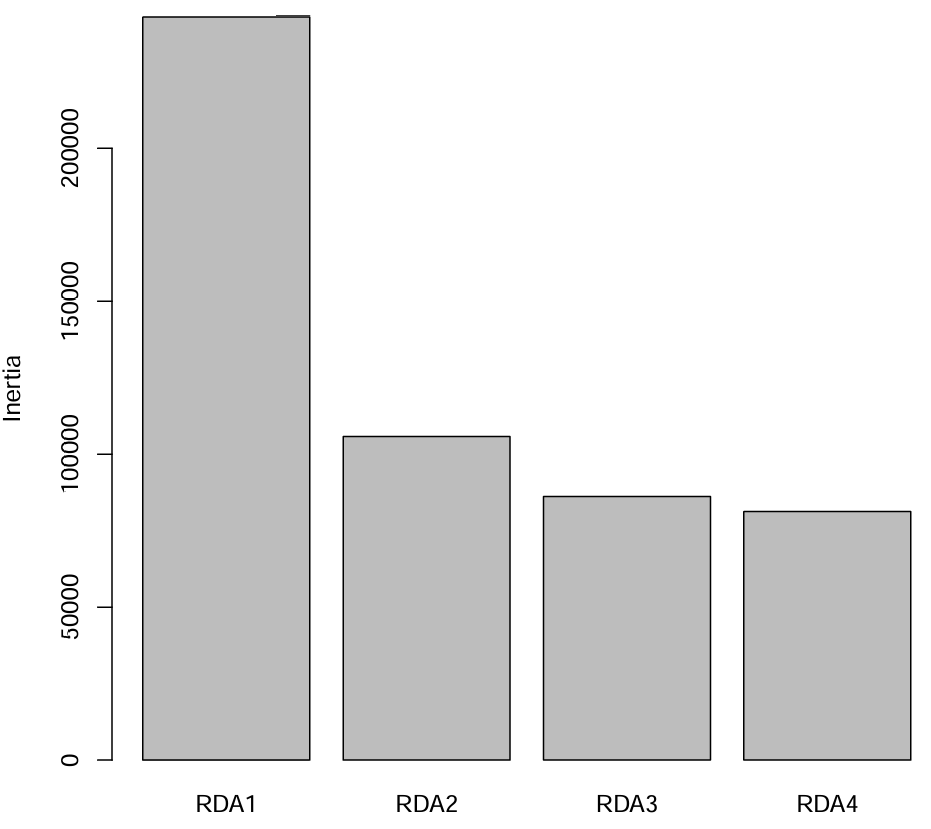


**Figure S6** RDA identified potentially adaptative SNPs. Variance explained by RDA axes.


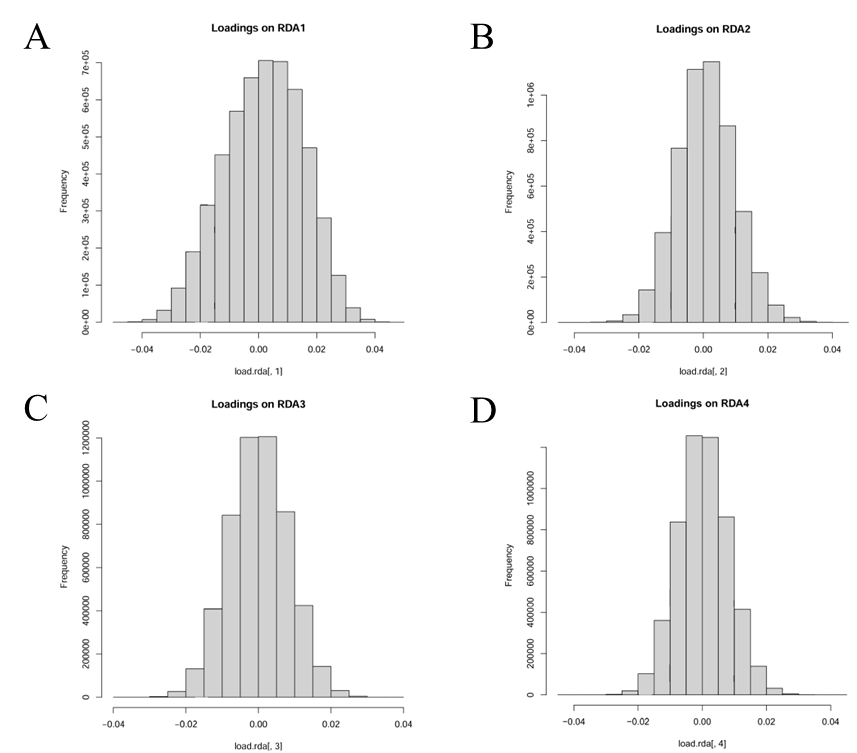


**Figure S7** RDA identified potentially adaptative SNPs. Frequency distribution of the first 4 RDAs.


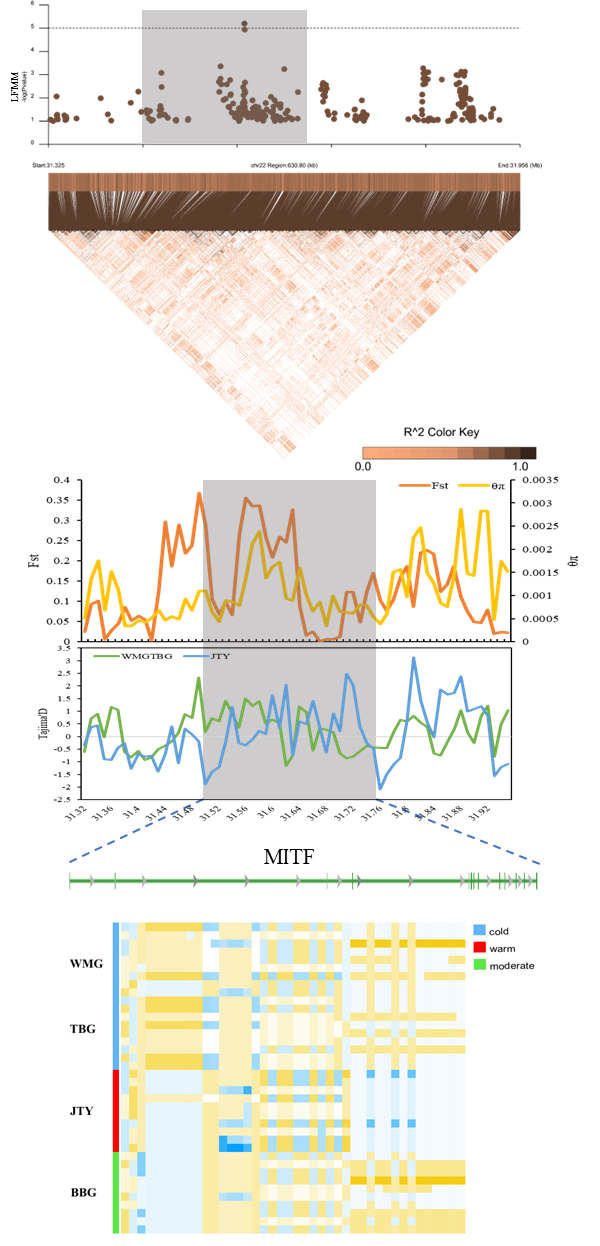


**Figure S8** Candidate adaptive genes analysis: MITF. Manhattan plots, LDblock plots showing candidate genes (MITF) associated with environmental temperature in cashmere goats. Fst, θπ ratio, and Tajima'D values are selected genes in cashmere goats. The green pattern diagram is the structural diagram of genes. Gene haplotype heatmap of selected genes in goats living at different environmental temperatures.


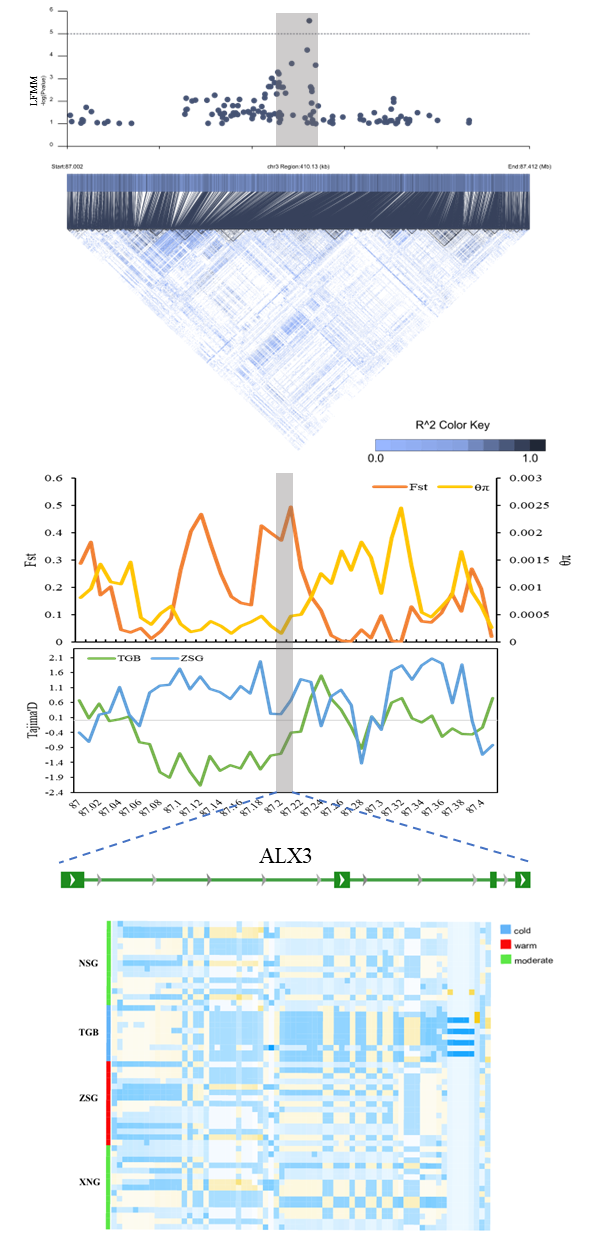


**Figure S9** Candidate adaptive genes analysis: ALX3. Manhattan plots, LDblock plots showing candidate genes (ALX3) associated with environmental temperature in dairy goats. Fst, θπ ratio, and Tajima'D values are selected genes in dairy goats. The green pattern diagram is the structural diagram of genes. Gene haplotype heatmap of selected genes in goats living at different environmental temperatures.


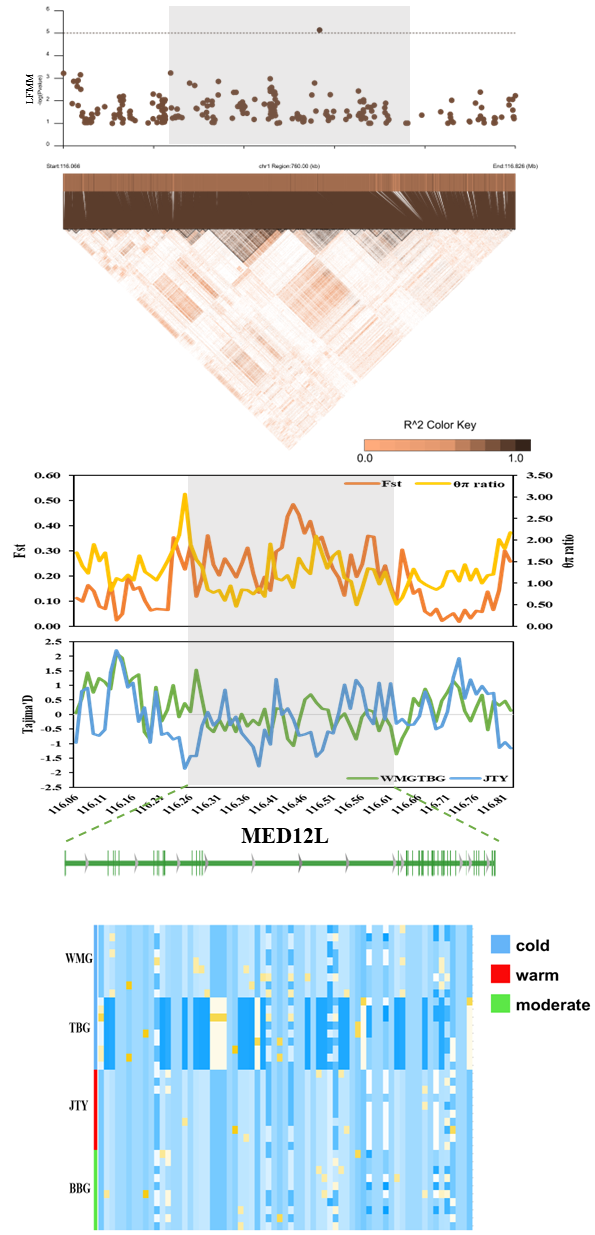


**Figure S10** Candidate adaptive genes analysis: MED12L. Manhattan plots, LDblock plots showing candidate genes (MED12L) associated with environmental temperature in cashmere goats. Fst, θπ ratio, and Tajima'D values are selected genes in cashmere goats. The green pattern diagram is the structural diagram of genes. Gene haplotype heatmap of selected genes in goats living at different environmental temperatures.


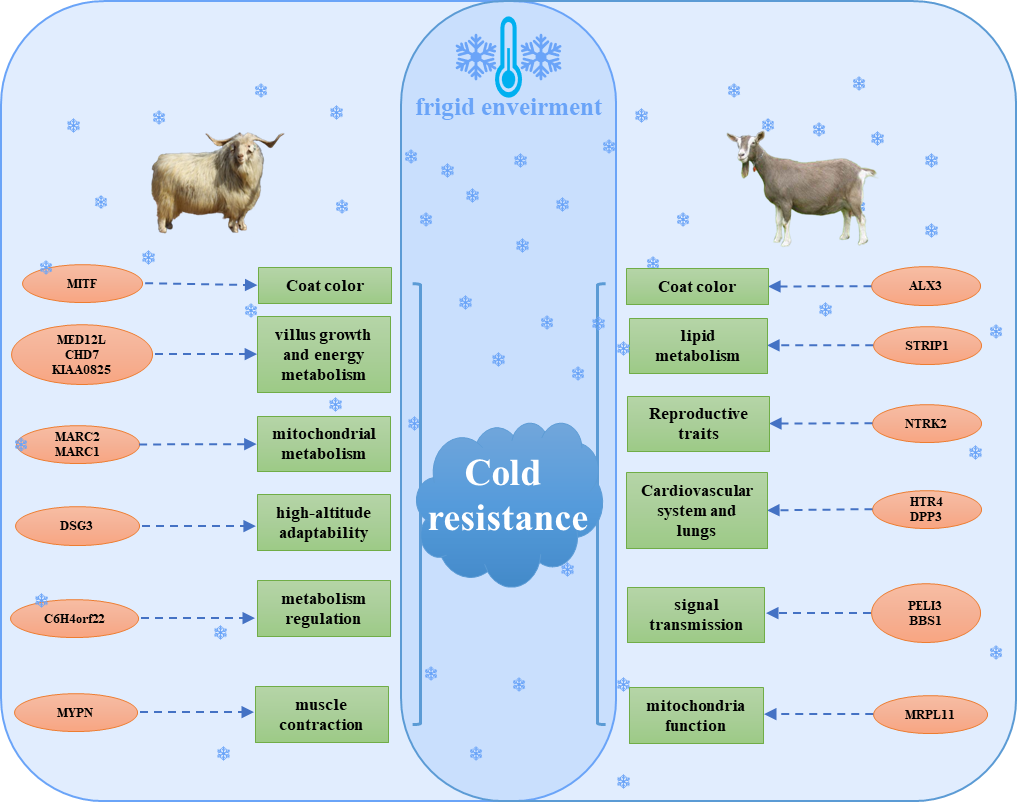


**Figure S11** Overview schematic of the study. Cashmere goats and dairy goats use related genes to exert different functions and jointly resist cold in frigid environments.
